# Refractory metabolic acidosis and acute abdominal compartment syndrome following Holmium Laser Enucleation of Prostate (HoLEP)

**DOI:** 10.2478/jccm-2025-0027

**Published:** 2025-07-31

**Authors:** Murugananth Nithiyananthan, Shitalkumar Sharad Shah, Aarthi Suhitharan, Suhitharan Thangavelautham

**Affiliations:** Singapore General Hospital, Singapore, Singapore; National Junior College, Singapore, Singapore

**Keywords:** HoLEP, metabolic acidosis, normal saline, hyperchloremia, abdominal compartment syndrome, continuous renal replacement therapy

## Abstract

**Introduction:**

Holmium Laser Enucleation of the Prostate (HoLEP) is a widely used minimally invasive surgical technique for benign prostatic hyperplasia (BPH), offering advantages such as reduced bleeding, shorter hospitalization, and elimination of TURP syndrome. However, complications related to fluid absorption and capsular perforation can still occur. We report a rare case of severe refractory metabolic acidosis and acute abdominal compartment syndrome (ACS) following HoLEP.

**Case Presentation:**

A 74-year-old male with diabetes and hypertension underwent HoLEP for a 180-ml prostate, during which 106 liters of normal saline irrigation were used over three hours. Intraoperatively, the patient developed sudden respiratory distress and hypotension, with arterial blood gas analysis revealing severe metabolic acidosis (pH 7.141, HCO_3_ 11 mEq/L, Cl 115 mEq/L), primarily due to excessive saline absorption and hyperchloremia. The patient required intubation, vasopressor support, and emergency dialysis due to worsening hemodynamic instability. Postoperative imaging revealed intra-abdominal fluid collection, which was drained percutaneously. After two days of intensive ICU management, the acidosis resolved, and the patient was successfully extubated.

**Conclusion:**

This is the first case highlighting the potential risks of normal saline absorption and the effect of capsular perforation, which caused ACS and refractory acidosis, and required CRRT due to the prolonged duration of HoLEP.

## Introduction

Holmium Laser Enucleation of the Prostate (HoLEP) is a state-of-the-art, minimally invasive surgical technique that has become the preferred treatment for benign prostatic hyperplasia (BPH), particularly in patients with larger prostates [1]. The laser energy used in HoLEP has cutting and coagulative properties, meaning that the small blood vessels injured during surgery are sealed off immediately, leading to a lower incidence of bleeding and intravascular fluid reabsorption compared to the conventional transurethral resection of prostate [2]. Known for its precision and efficiency, HoLEP offers significant advantages over traditional procedures, including faster recovery, shorter hospital stays, and minimal risk of complications [3].

Despite its safety profile, complications can still arise. The most common complication of HoLEP is transient stress urinary incontinence, occurring in up to 25% cases, though persistent incontinence is rare (<3%) [4]. Urinary tract infections (4–12%) and urethral stricture or bladder neck contracture (1–5%) have also been observed [5,6]. Significant bleeding requiring transfusion is uncommon (<3%), while clot retention and urinary retention occur in 1–2% and 2–6% of patients, respectively [7].

During HoLEP, continuous irrigation is necessary to maintain a clear surgical field and to cool the laser tip. Historically, normal saline has been the irrigation fluid of choice. Severe complications, such as fluid overload, hyperchloremic metabolic acidosis, and acute kidney injury (AKI), are rare but may occur during lengthy procedures involving high irrigation volumes [8] in high-risk patients (large prostate >100 ml, cardiac/renal comorbidities). Capsular perforation (1–2%) and bladder injury (<1%) are rare but more likely with large prostate volumes or prolonged surgeries [5]. Perforation of the prostatic capsule leads to massive intravascular absorption, directly via the prostatic venous sinuses and indirectly via the absorption of leaked irrigation fluid into the perivesical space and peritoneal cavity [9]. Furthermore, fluid leakage from the urinary tract due to the pressure gradient may potentially lead to abdominal compartment syndrome (ACS).

Here, we present a rare, yet critical case of severe intraoperative metabolic acidosis triggered by irrigation fluid reabsorption and ACS, that ultimately necessitated ICU admission and emergency dialysis.

## Case presentation

A 74-year-old male with a history of diabetes mellitus, hypertension, and Benign Prostatic Hyperplasia (BPH) presented with lower urinary tract symptoms, including nocturia, frequency, and voiding difficulties. The estimated prostate volume on the preoperative radiological scan was 180 ml. He was scheduled for elective HoLEP at Sengkang General Hospital, Singapore.

The procedure was performed under a combined spinal-epidural (CSE) anesthesia approach. Spinal anesthesia was achieved with 2.2 ml of 0.5% intrathecal heavy bupivacaine and 15 mcg of fentanyl, which were administered through the 27G pencil point spinal needle via the 18G Tuohy needle, followed by the insertion of epidural catheter. A sensory blockade up to the T6 dermatome was achieved, with a modified Bromage score of 4. The patient was sedated using target-controlled infusion (TCI) of propofol, with an effect-site concentration ranging from 0.5 to 1 μg/mL. He remained hemodynamically stable during the first two hours of surgery.

Two hours into the procedure, the patient had mild abdominal discomfort, which was attributed to a regression of the sensory level to T10. An epidural topup of 5 mL of 0.5% ropivacaine was administered to maintain adequate analgesia. At the three-hour mark of the procedure, the patient reported abdominal discomfort with a sensory blockade at T8. In response, an additional 5 mL of 0.5% ropivacaine was administered via the epidural catheter with good symptomatic relief. However, within 30 minutes he developed severe abdominal pain, acute respiratory distress, and hemodynamic instability with Blood pressure (BP) of 85/46 mmHg and Heart rate (HR) of 110 beats per minute.

At this point, a total of 144 grams of prostate tissue were resected using 106 liters of normal saline for irrigation. BP was maintained with intermittent boluses of phenylephrine and ephedrine, totaling 200mcg and 10mg respectively. On examination there was abdominal distension with minimal air entry to both lower zones of the lungs. The surgeon, who was informed about the deterioration, acknowledged that there was a possibility of prostatic capsular perforation. The surgeon was advised to achieve hemostasis and complete the procedure as soon as possible in view of the patient’s clinical deterioration.

Arterial blood gas analysis showed severe metabolic acidosis characterized by hyperchloremia and lactic acidosis (Table 1). Central venous access and invasive blood pressure monitoring were established. The patient was started on noradrenaline continuous infusion, intubated, and mechanically ventilated. Hemostasias was achieved using bipolar coagulation, a suprapubic catheter was inserted for diversion of urine, and the surgery was completed within 30 minutes of intubation. The patient was promptly transferred to the ICU for respiratory support and management of severe acidosis.

**Table 1.** Arterial Blood Gas (ABG) Trends Demonstrating Severe Metabolic Acidosis Following HoLEP and Resolution Post-CRRT.

**ABG parameter**	**Intraop**	**Day 0**	**Day 1 AM**	**Day 1 PM**	**Day 2**	**Day 3**
FIO_2_ (%)	1	0.5	0.3	0.3	0.3	0.21
pH	7.102	7.141	7.211	7.398	7.412	7.441
pCO_2_ (mmHg)	42.2	37.6	35.9	35.8	35.6	33.9
pO_2_ (mmHg)	278	151.6	79.4	82.8	98.9	78.6
Base Excess (mEq/L)	−16	−15.6	−12.8	−2.8	−2.1	−1.2
Bicarbonate (mEq/L)	13.2	12.7	14.5	20.6	22.6	23.4
Sodium (Na^+^, mEq/L)	141	136	139	138	139	139
Potassium (K^+^, mEq/L)	4.3	6.0	4.5	4.0	3.9	3.5
Chloride (Cl^−^, mEq/L)	115	115	113	109	110	105
Lactate (mmol/L)		3.3	3.2	4.8	2.7	1.7
Glucose (mmol/L)	12.1	14.9	15.9	6.6	8	7.7
Anion Gap (mEq/L)	12.8	8.3	11.5	8.4	6.4	10.6

Day 1 AM = Morning values; Day 1 PM = Evening values.

A computed tomography (CT) scan of the abdomen and pelvis done upon ICU admission confirmed that the prostatic capsular perforation caused extensive intra- and extraperitoneal fluid collections (Figures 2 and 3). Intravesical pressure measured in the ICU was 23 mmHg, confirming the diagnosis of abdominal compartment syndrome (ACS). A percutaneous intra-abdominal drain was inserted under ultrasound guidance at bedside, and it drained 1300ml of hemoserous fluid within the first two hours since insertion.

**Fig. 1.**
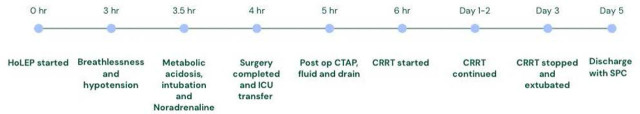
Timeline of Clinical Events in HoLEP Case. CTAP – Computed Tomography of Abdomen and Pelvis. SPC – Suprapubic Catheter.

**Fig. 2.**
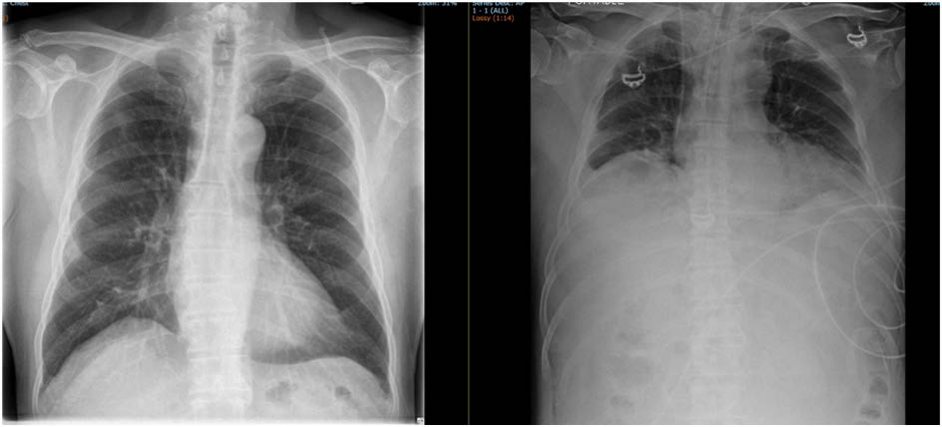
Comparison of Preoperative and Postoperative Chest X-rays in the patient with Abdominal Compartment Syndrome following HoLEP. The left image shows a normal preoperative chest X-ray, while the right image demonstrates postoperative abdominal distension and diaphragmatic splinting with reduced lung fields.

**Fig. 3.**
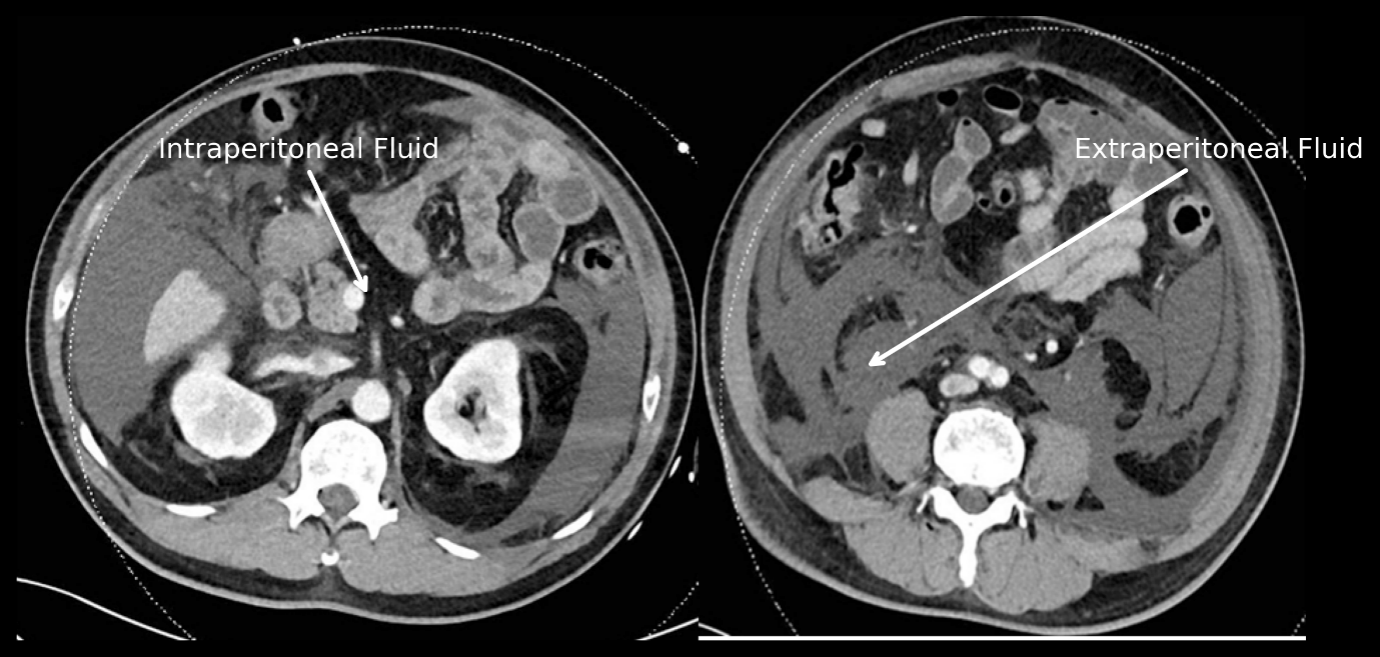
Abdominal CT done postoperatively on ICU admission showing fluid seepage into Extraperitoneal and Intraperitoneal Compartments. The left panel demonstrates intraperitoneal fluid (white arrow on the left) around the mesentery and bowel loops, while the right shows extraperitoneal fluid (white arrows on the right) in the retroperitoneal and perivesical spaces.

In the ICU, the patient was initiated on continuous renal replacement therapy (CRRT) due to refractory metabolic acidosis and hyperkalemia (Table 1). After two days of CRRT, the patient was successfully extubated on Day 3 of ICU admission and was discharged to ward on Day 5 with a suprapubic catheter planned to be kept for 4–6 weeks.

## Discussion

Our patient’s clinical deterioration is due to dual pathology: abdominal compartment syndrome (ACS) and severe hyperchloremic metabolic acidosis. ACS was confirmed with intra-abdominal pressure of 23 mmHg and the development of acute kidney injury and hypoxic respiratory failure due to diaphragmatic splinting [10].

Reported cases of severe complications of HoLEP can be summarized into two phenotypes. The first type is severe metabolic acidosis due to excessive fluid absorption. These patients exhibit clinical evidence of fluid overload, such as upper airway or facial edema and pulmonary edema, which manifests as orthopnea, hypoxia and pink frothy sputum. [11,12]. Metabolic acidosis and pulmonary edema typically respond to furosemide therapy within few hours of ICU admission. The second phenotype of severe complication is due to ACS caused by bladder or prostatic capsular perforation, which is presented with abdominal distention, raised airway pressures and hemodynamic instability [13]. To our knowledge, our patient is the first reported case of ACS and severe metabolic acidosis after HoLEP that required positive pressure ventilation, vasopressor support, and CRRT in ICU.

The average operative time for HoLEP is typically 60–90 minutes for moderate-sized prostates and up to 120–150 minutes for very large prostates (>150–200 cc) [14,15]. A multicenter study by Schmidt et al. reported a mean duration of 81 minutes, with longer surgeries associated with higher complication rates and prolonged catheterization [16]. Hartung et al. found that procedures exceeding 150 minutes were independently linked to higher incidences of fluid-related complications, urinary retention, and metabolic disturbances, particularly in high-risk patients [17].

Our patient did not have any evidence of intravascular fluid overload, despite having risk factors such as prolonged surgery, a large prostate size, and a large amount of irrigation fluid used. This could be due to the capsular perforation causing the fluid to accumulate in the abdomen rather than being absorbed into the vascular compartment. AKI caused by ACS may have worsened his metabolic acidosis by abolishing the renal compensation.

Richter et al. [13] reported a case of ACS during HoLEP in an intubated patient. However, that patient did not develop AKI or severe metabolic acidosis. This difference may be explained by that patient’s elevated venous pressures associated with positive pressure ventilation, which may reduce the pressure gradient between the bladder filled with irrigation fluid and the exposed prostatic venous sinuses, thereby limiting intravascular fluid absorption, compared to our patient who was breathing spontaneously.

Normal saline is isotonic with plasma, cost-effective, and compatible with bipolar electrosurgical systems. However, its high chloride content may predispose patients to hyperchloremic metabolic acidosis if absorbed in significant volumes, and its lack of buffering capacity may exacerbate acid-base disturbances during prolonged procedures [18]. Chloride-induced acidosis can promote inflammation by increasing renal vasoconstriction and tubular injury through chloride-mediated thromboxane release, which exacerbates inflammatory cytokine production and oxidative stress, compromising microvascular perfusion, impairing oxygen delivery, and causing tissue damage [19]. On the other hand, balanced crystalloids, by maintaining a more physiological pH and reducing hyperchloremia, mitigate these inflammatory pathways, preserving endothelial function and reducing pro-inflammatory cytokine release, ultimately minimizing tissue injury [20].

Preventing severe complications such as hyperchloremic acidosis during HoLEP necessitates a proactive approach centered on early risk identification and intraoperative vigilance. Patients with large prostate volumes (>100 cc) and pre-existing cardiac or renal comorbidities should be identified during preoperative evaluation. Intraoperative strategies include minimizing surgical duration, considering staged procedures in complex or prolonged cases, meticulously monitoring irrigation fluid volume, and continuously surveilling electrolyte and hemodynamic parameters. The use of regional anesthesia facilitates early recognition of complications through patient-reported symptoms, while timely arterial blood gas (ABG) analysis enables prompt identification of acid-base disturbances. In the event of capsular or bladder perforation, effective communication between the surgical and anesthetic teams is critical, with consideration being given to the termination of the procedure. Intermittent monitoring of bladder pressures, insertion of pelvic drains, and administration of diuretics should be considered when the surgery is unable to be terminated imminently. Where feasible, if normal saline is used for irrigation, it should be replaced with balanced crystalloids such as Plasma-Lyte or Ringer’s lactate solution to reduce the risk of hyperchloremic acidosis. In our case, even after the confirmation of capsular perforation, we were unable to use balanced crystalloids as irrigation fluid intraoperatively due to institutional limitations. The only balanced crystalloid solution available in our operating room was Ringer’s lactate solution packed in 500 ml plastic bottles, which was not feasible to administer in the irrigation system. It is recommended that the operation theatre bladder irrigation protocol be reviewed regularly, with designated responsibility assigned to the anaesthesia and urology teams. Additionally, steps should be taken to ensure the availability of Ringer’s lactate solution in 3-liter soft bags as an alternative irrigation fluid.

## Conclusion

This case highlights a rare but serious complication of hyperchloremic acidosis with ACS after HoLEP due to prolonged surgery, excessive irrigation, and capsular perforation. Early risk-identification, intraoperative vigilance, and prompt intervention are critical for the prevention of such complications. In the event of capsular perforation during HoLEP, collaborative intraoperative care, consideration of staged procedures, and the use of balanced crystalloids as irrigation should be considered to mitigate the severe complications.
